# Healthcare Professional Preferences for Prescribing Artemisinins and Quinine for Malaria in Burundi

**DOI:** 10.24248/eahrj.v5i2.670

**Published:** 2021-11-15

**Authors:** Aîné-Ernest Niyonkuru, Eric McLaughlin, Gregory Heath, Sonia Inamuco, Hillary Topazian, Mike Davis

**Affiliations:** a Hope Africa University (Burundi); b The University of Tennessee at Chattanooga; c UNC-Chapel Hill: University of North Carolina at Chapel Hill

## Abstract

**Background:**

Malaria is a significant cause of morbidity and mortality throughout the world and particularly sub-Saharan Africa. The World Health Organization and many national bodies, including Burundi, recommend artemisinin-based therapy as first-line treatment for uncomplicated and severe malaria. Implementing this recommendation requires healthcare professionals' acceptance of this treatment as the optimal choice.

**Methods:**

A survey was conducted among Burundian healthcare professionals from June to September 2017 to assess prescribing preferences regarding artemisinins versus quinine for treating malaria. Healthcare professionals were surveyed from 32 health facilities in 10 provinces. Respondents included both physicians and nurses who provided responses about their antimalarial treatment preferences for a variety of clinical scenarios. Comparisons among healthcare professionals, their level of training, work setting, and length of work experience were examined using a series of stratified analyses, where the Pearson Chi-square statistic and odds ratios with 95% confidence intervals were calculated.

**Results:**

Respondents included 101 doctors and 196 nurses. Seventy-nine percent of respondents worked in hospitals, while 58% had more than 5 years of work experience. Although 94% of respondents correctly identified artemisinin-based treatment as first-line therapy according to the national protocol, 24-40% of respondents preferred the use of quinine in various hypothetical clinical scenarios. Overall, nurses were at greater odds of preferring quinine versus artemisinins compared with physicians. Availability of artemisinins was associated positively with artemisinin preference. These results did not vary by duration of work experience.

**Conclusions:**

Though knowledge of artemisinin-based therapy was recognised by the majority of respondents as the recommended antimalarial treatment, a high proportion of Burundian healthcare professionals, especially nurses, prefer using oral and IV quinine in a number of clinical scenarios. These findings identify a significant barrier to the satisfactory implementation of a life-saving treatment in accordance with national and international recommendations.

## BACKGROUND

**FIGURE 1:**
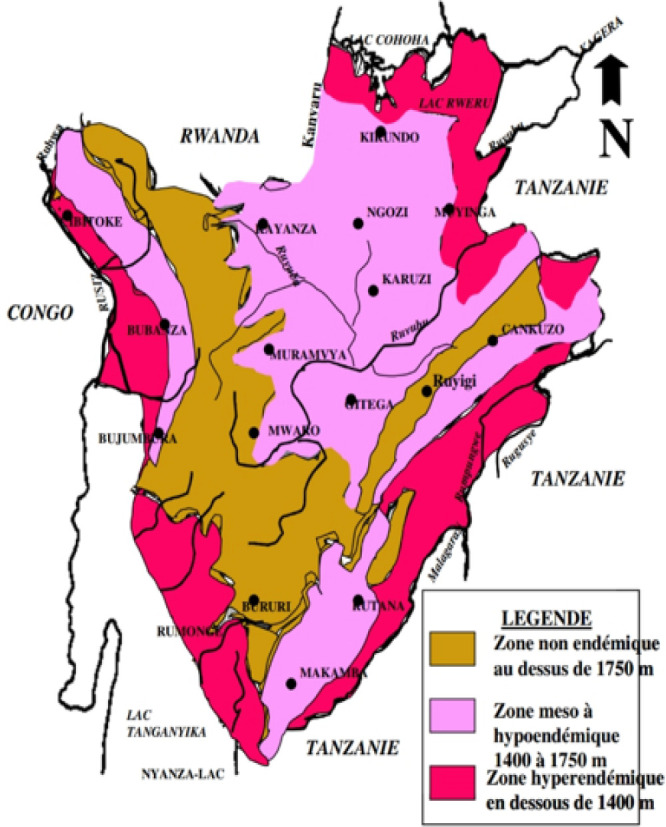
Geographic Zones for Malaria in Burundi

Malaria is a significant cause of morbidity and mortality throughout the world, and particularly in sub-Saharan Africa.^1^ In Burundi, malaria remains the leading cause of morbidity and mortality (see Figure 1).^2^ One of the largest advances in treatment in recent years is the introduction of artemisinin-based therapy. Most countries in Africa have been recommending artemisinin combination therapy (ACT) for first-line treatment of uncomplicated malaria since at least 2005, with Burundi adopting this recommendation in 2003.^3^ More recently, the South East Asian Quinine Artesunate Malaria Trial (SEAQUAMAT)for adults and the Artesunate versus quinine in the treatment of severe falciparum malaria in African children (AQUAMAT) trialfor children demonstrated decreased mortality for intravenous artesunate versus quinine in the treatment of severe falciparum malaria, a finding further supported by a 2012 Cochrane review.^4,5,6^ The World Health Organization (WHO) Guideline for the Treatment of Malaria has adopted artemisinin-based treatment for both categories since 2010.^7^ An African logistical study showed that artesunate is better, cheaper, and easier to administer than its longstanding forerunner quinine.^8^

Despite the enormous benefit of this evidence-based recommendation, the WHO World Malaria Report 2019 mentions that only 48% of febrile children in the public sector of sub-Saharan Africa who sought care were given an antimalarial drug, and that only 80% of those given an antimalarial were given an ACT.^1^ Though progress continues to be made, obstacles to complete implementation merit analysis. Successful implementation depends on a multiplicity of factors, including availability and acceptance by healthcare professionals (HCP).^9^

Regarding uncomplicated malaria in Burundi, sulfadoxine-pyrimethamine (SP) was officially replaced by artesunate-amodiaquine (AS-AQ) in november 2003, though quinine use is still widespread.^3^ In terms of availability, a Burundian study in 2011 showed that ASAQ was present in 88% of public sector venues.^10^ More recently, a 2019 national guideline changed the first-line ACT to artemether-lumefantrine (A-L), which has recently become widely available in Burundi. Second-line therapy is dihydroartemisinin-piperaquine, with oral quinine moving from second-line therapy to being used only in cases where there are contraindications to ACT (Table 1).^11^

**TABLE 1:** Burundi National Guideline Recommendations for Treatment of Malaria

	First-line therapy	Second-line therapy
**2012 Guideline (at time of study)**		
Simple Malaria	Artesunate-Amodiaquine	Quinine + Clindamycin*
Severe Malaria	Injectable Artesunate	Injectable Quinine
**2019 Guideline (current)**		
Simple Malaria	Artemether-Lumefantrine	Dihydroartemisinin-Piperaquine**
Severe Malaria	Injectable Artesunate	Injectable Quinine

* During this period, Clindamycin was not routinely available, and thus quinine monotherapy was common

** Quinine is described as an alternative only in cases of contraindications to ACT

Note: In the 201 2 and 2019 guidelines, Quinine + Clindamycin is recommended for the first trimester of pregnancy

Acceptability and preference of these antimalarial drugs by health-care professionals and the population has been evaluated in a variety of heterogeneous studies across sub-Saharan Africa. A Rwandan study of non-artemisinin antimalarials found HCP non-compliance to be associated with ignorance of the protocol, doubt of efficacy, and fear of adverse effects.^12^ In two Cameroonian studies regarding implementation of AS-AQ, HCPs' hesitancy was due to adverse effects of amodiaquine, lack of availability, and doubt of efficacy.^13,14^ Two Burkina Faso studies also showed poor compliance to ACTs for uncomplicated malaria, despite adequate knowledge of national protocol recommendations, citing as reasons adverse effects and availability.^15,16^ A Kenyan study showed quinine prescription persisting particularly in children over 5 years and adult populations.^17^

In regard to severe malaria, since 2012, Burundi's national protocol has recommended IV artesunate as first-line therapy with IV quinine as a second-line alternative when IV artesunate is unavailable. We were not able to find current availability data and IV quinine use remains widespread.^18^ Preference for treating severe malaria with IV quinine was elsewhere seen in a 2016 Congolese study where less than 2% received IV artesunate.^19^ A 2015 Sudanese study also found overuse of IV quinine instead of oral ACTs for patients that had no criteria for severe malaria.^20^

In light of the paucity of studies about HCP preferences towards any artemisinin-based therapy in our region of Africa, and in particular of studies that evaluate such preferences for injectable artesunate for severe malaria, our study sought to address these topics in the context of the country of Burundi. If HCP preferences diverge from national and international recommendations, identification of this divergence could highlight an important gap in the pathway from correct knowledge to correct implementation.

## METHODS

### Study Area and Period

This cross-sectional study was performed between June and September 2017, roughly 5 years after the 2012 national guideline instituted injectable artesunate for severe malaria. In an effort to approach greater generalizability of our findings across Burundi, we elected to perform convenience sampling of facilities in ten of the eighteen provinces of Burundi, as well as convenience sampling of HCPs at those facilities. Five of the provinces (Bujumbura Mairie, Bubanza, Cibitoke, Bururi, and Rumonge) are in the hyper-endemic zone for malaria, and the other five provinces (Gitega, Kayanza, Ngozi, Mwaro, and Muyinga) are in the hypo-endemic zone, which was significantly affected by the malaria epidemic announced in March 2017.^21^ Seventeen hospitals were surveyed (12 public, 1 private, and 4 partnered faith-based). Fifteen nursing-staffed health centers were surveyed (8 public, 6 private, and 1 partnered faith-based).

### Study Population

Assuming an alpha = 0.5 and a baseline 0.5 acceptance rate given a lack of previous study results in the region, we estimated a sufficient sample size of 98 physicians and 98 nurses to detect a change of 0.17 between the two groups. Given that physicians and nurses form the backbone of malaria diagnosis and treatment in the Burundian health system, this study uses the term healthcare professionals to refer to these two groups, though the general definition includes those trained to work in a broader range of health fields. All physicians and nurses present at a visit by the principal investigator to the above facilities were given the opportunity to complete the survey, including both consultant-level and generalist physicians, as well as nurses of all qualifying professional levels. A convenience sample of 297 respondents was obtained, including 101 physicians (20 consultants and 81 generalists) and 196 nurses, 43 of which were registered nurses (level A0) and 153 of which were enrolled nurses (level A2 or A3).

**FIGURE 2:**
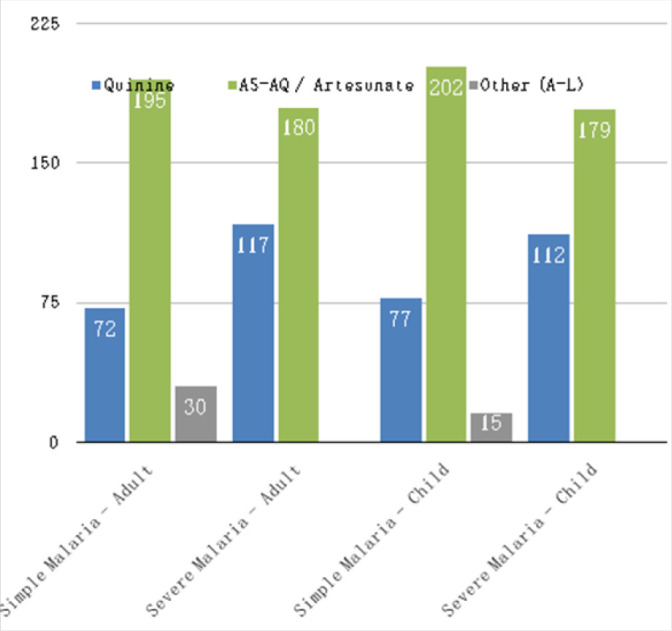
Healthcare Professionals' Teatment Preferences for Simple and Severe Malaria in Adults and Children

**FIGURE 3:**
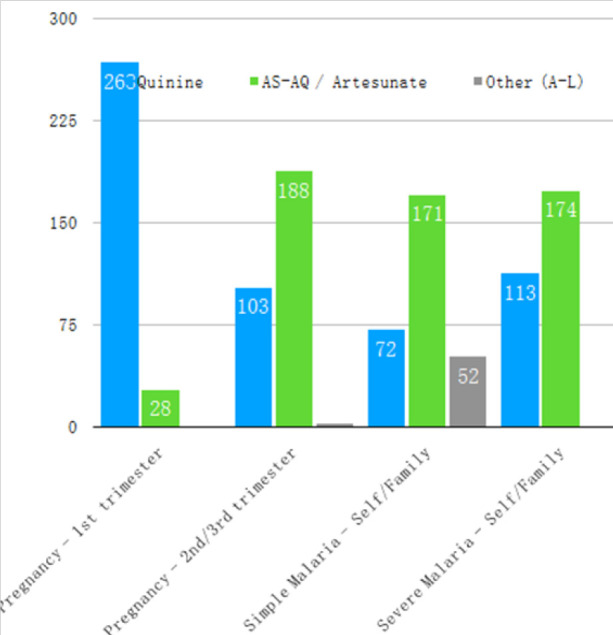
Healthcare Professionals' Treatment Preferences for Malaria during Pregnancy and Simple/Severe Malaria for Self/Family Member

### Survey Methodology

A written questionnaire designed by the principal investigator was administered in French directly by the principal investigator, with the possibility of asking clarifying questions in Kirundi if desired by the participant.

Respondents first identified their place of work (hospital or health center) followed by their profession (physician or nurse, with corresponding level). They also self-reported their professional experience as greater than or less than 5 years. Each respondent then reported the availability of intravenous (IV) artesunate, IV quinine, oral AS-AQ and oral quinine in their place of work. Next, respondents answered 6 questions about their preferred anti-malarial to prescribe in various scenarios. The 3 categorical response options were artesunate (or AS-AQ in the case of uncomplicated disease), quinine (including oral quinine in the case of uncomplicated disease), and other choice (in the rare cases of this being chosen, it corresponded to oral A-L). Respondents were then asked which antimalarial they would prefer to receive if they or a close member of their family were diagnosed with uncomplicated or severe malaria. These scenarios were followed by a question about which antimalarial causes more side effects, with the same categorical choices. Finally, their knowledge of the current Burundian recommendation was assessed by asking them to identify the first-line antimalarial according to the national protocol.

### Data Analysis

All survey responses were transferred into a data spreadsheet with respondents coded by number. The survey responses were then uploaded from the data spreadsheet into SPSS (IBM, Version 25.0) for data analysis. All responses were categorical. Through a series of stratified analyses, specifically stratified by healthcare professionals (i.e. physicians and nurses), years of healthcare service, and availability of artesunate and ASAQ at the respondent's facility, we examined associations with the scenario outcomes. Measures of association for each of these stratified analyses used the Pearson chi-square test (*p<0.05*), odds ratios (OR) and 95% confidence intervals (CI).

### Ethics and Consent

Analysis of the survey data and study protocol were approved by the University of Tennessee at Chattanooga (USA) Institutional Review Board (IRB #18-167). All respondents provided consent by completing the survey instrument. No personal identifiers were collected.

## RESULTS

Of the total 297 HCPs surveyed, 101 (34%) were physicians and 196 (66%) were nurses. Two hundred thirty five (79%) respondents worked in hospitals, and 62 (21%) in nurse-run health centers. One hundred twenty four (42%) respondents had less than 5 years' healthcare work experience (Table 2).

**TABLE 2:** Characteristics of Healthcare Professional Respondents

Characteristic	All Healthcare Professionals	Physicians	Nurses
**Professional training**	297 (100%)	101 (34%)	196 (66%)
Consultant Physician	20 (7%)	20 (20%)	—
Generalist Physician	81 (27%)	81 (80%)	—
Registered Nurse (AO level)	43 (14.5%)	—	43 (22%)
Enrolled Nurse (A2/A3 level)	153 (51.5%)	—	153 (78%)
**Place of work**			
Hospital (79% of total)	235 (100%)	101 (43%)	134 (57%)
Public Hospital	141 (60%)	61 (60%)	80 (60%)
Private Hospital	31 (13%)	16 (16%)	15 (11%)
Partner/Faith-Based	63 (27%)	24 (24%)	39 (29%)
**Health Centre (21% of total)**	62 (100%)	0 (0%)	62 (100%)
Public Health Centre	43 (69%)	0 (0%)	43 (69%)
Private Health Centre	14 (23%)	0 (0%)	14 (23%)
Partner/Faith-Based	5 (8%)	0 (0%)	5 (8%)
**Years of healthcare experience**			
Less than 5 years (42% of total)	124 (100%)	47 (38%)	77 (62%)
5 years or more (58% of total)	173 (100%)	54 (31%)	119 (69%)

### Availability of antimalarials

All healthcare professionals indicated that oral quinine was available at their place of work, with 281 (95%) respondents reporting the availability of IV quinine (Table 3). Artemisinin-based therapy was less available, with 203 (68%) HCPs reporting the availability of oral AS-AQ, and 187 (63%) reporting access to IV artesunate. These therapies were exclusively available at public or partnered faith-based facilities. Excluding private facilities, 203 of 252 respondents (81%) reported availability of AS-AQ, and 187 (74%) reported availability of IV artesunate.

**TABLE 3:** Availability of Antimalarials

	Public	Private	Partner/Faith-Based	TOTAL
**Hospitals (n=235)**				
AS-AQ (PO)	107/141 (75.9%)	0/31 (0.0%)	48/63 (76.2%)	155/235 (66.0%)
Artesunate (IV)	107/141 (75.9%)	0/31 (0.0%)	48/63 (76.2%)	155/235 (66.0%)
Quinine (PO)	141/141 (100%)	31/31 (100%)	63/63 (100%)	235/235 (100%)
Quinine (IV)	141/141 (100%)	31/31 (100%)	63/63 (100%)	235/235 (100%)
**Health Centres (n=62)**				
AS-AQ (PO)	43/43 (100%)	0/14 (0.0%)	5/5 (100.0%)	48/62 (77.4%)
Artesunate (IV)	32/43 (74.4%)	0/14 (0.0%)	0/5 (0.0%)	32/62 (51.6%)
Quinine (PO)	43/43 (100%)	14/14 (100%)	5/5 (100.0%)	62/62 (100%)
Quinine (IV)	32/43 (74.4%)	14/14 (100%)	0/5 (0.0%)	46/62 (74.2%)
**All Respondents (n=297)**				
AS-AQ (PO)	150/184 (81.5%)	0/45 (0.0%)	53/68 (77.9%)	203/297 (68.4%)
Artesunate (IV)	139/184 (75.5%)	0/45 (0.0%)	48/68 (70.6%)	187/297 (63.0%)
Quinine (PO)	184/184 (100%)	45/45 (100%)	68/68 (100%)	297/297 (100%)
Quinine (IV)	173/184 (94.0%)	45/45(100%)	63/68 (92.6%)	281/297 (94.6%)

### Professionals' antimalarial preferences and beliefs about side effects

Two hundred seventy nine (94%) HCPs surveyed correctly identified artemisinin-based therapy as the recommended first-line therapy according to the Burundian national protocol. However, when presented with a variety of clinical scenarios, many of these HCPs still preferred quinine to artemisinin-based therapy (Figures 2 and 3; note that there was a 0 to 3% non-response rate, accounting for slightly varying total responses). This finding was more consistent for the three scenarios associated with severe malaria than for the clinical scenarios associated with simple malaria. For example, 72 (24%) respondents preferred quinine for simple malaria in adults, compared to 117 (39%) for severe malaria (*P<.01*). Similar results were found for pediatric cases (77 or 26% preferred quinine for simple malaria, and 112 or 38% for severe malaria, *P=.01*) as well as treatment choice if the respondent or their family member had malaria (72 or 24% preferred quinine for simple malaria versus 113 or 38% for severe malaria, *P=.02*).

Additional observations identified 268 (90%) respondents who preferred quinine for the first trimester of pregnancy (in accordance with the national protocol) versus 103 (35%) for the second and third trimesters (contrary to the national protocol but similar to other scenarios surveyed). 198 (67%) respondents believed that quinine causes more side effects than AS-AQ/Artesunate.

Stratified subgroup analyses showed significant differences between doctors and nurses in all three scenarios involving severe malaria, with physicians at greater odds than nurses of preferring intravenous artesunate (in accordance with the national protocol) than intravenous quinine for adults (OR 1.50; 95% CI, 1.3 to 1.8), for children (OR 1.45; 95% CI, 1.2 to 1.7), and for self or a family member (OR 1.56; 95% CI, 1.3 to 1.9, see Table 4 for details).

**TABLE 4:** Treatment Preference for AS-AQ or Artesunate by Profession (n=297)

Treatment of Choice	Physicians preferring AS-AQ or Artesunate n=101	Nurses preferring AS-AQ or Artesunate n=196	Odds Ratio (95% Cl)	*Significance*
Simple malaria - adult	71 (76%)	123 (71%)	1.29 (0.686, 2.45)	p=.4242*
Severe malaria - adult	78 (77%)	101 (52%)	1.50 (1.26, 1.78)	p=.0001
Simple malaria-child	74 (76%)	128 (71%)	1.22 (0.653, 2.31)	p=.5251
Severe malaria - child	77 (78%)	103 (54%)	1.45 (1.23, 1.72)	p=.0001
Simple malaria - self/family	54 (74%)	117 (69%)	1.28 (0.688, 2.38)	p=.4347
Severe malaria - self/family	79 (79%)	95 (51%)	1.56 (1.31, 1.85)	p=.0001

* Except when artesunate is not available – Nurses are at greater odds of indicating Quinine as preferred treatment +based on the Pearson Chi Square Statistics+

However, there were no significant differences between physicians and nurses for the three scenarios involving simple malaria, which compared oral AS-AQ and oral quinine (*P=.4* for adults, *P=.5* for children, *P=.4* for self or family member). Stratified subgroup analyses of HCPs with more or less than five years' work experience showed no statistically significant differences in any antimalarial preferences (*P=.99* for simple malaria and *P=.37* for severe malaria).

### Associations between antimalarial preference and availability

When treatment preferences were analyzed based on availability of AS-AQ in the respondent's place of work, availability of AS-AQ was associated with respondents' preference for using it (*P=.003*). A similar association was found when comparing the availability of IV artesunate and the health-care professionals' preference for using this medicine (*P=.007*).

## DISCUSSION

Acceptance by the healthcare professionals who will put a national protocol into practice is one of many essential elements of successful implementation. This study shows that knowledge of the national protocol in Burundi is excellent, but diverges significantly from the practical preferences in a sizable proportion of Burundian HCPs surveyed. This gap between knowledge and practical preference suggests an acceptance problem of the recommended antimalarials by at least part of the workforce that is responsible for implementing the protocol. As a particular example of this phenomenon, 268 (90%) respondents' preferences were in agreement with the national protocol in the one scenario that recommends quinine therapy (first trimester of pregnancy). However, later in pregnancy, when artemisinin-based therapy is recommended, only 188 (63%) of respondents' preferences agreed with the protocol, a level of agreement very similar to the other non-pregnancy scenarios.

In our study, this gap between knowledge and practical preference was significantly more pronounced in the nursing workforce compared to physicians. This is especially consequential since nurses are prescribing these antimalarials throughout the country's health centers. Years of health care experience does not appear to modify this distinction. Both of these findings were contrary to a Tanzanian study which found longer work experience and non-doctor prescribers associated with increased ACT usage.^22^

What are the root causes of this problem of acceptance for a medicine whose efficacy has an excellent evidence base and whose role at the front of the national protocol is nearly universally known? To be sure, further studies are needed to explore this question more directly.

One might posit that the amodiaquine portion of AS-AQ is a primary reason for hesitation in artemisinin-based therapy. This is supported by the HCPs who voluntarily wrote in A-L as “other” when asked their preferences. However, this study clearly shows that the significant differences between nurses and doctors resides in the treatment of severe malaria, where amodiaquine is not implicated. Thus, this problem could persist even with the replacement of AS-AQ by A-L in the 2019 national protocol.

As shown above, the statistically significant correlation between both AS-AQ and artesunate availability and treatment preference for these recommended medicines suggests that unfamiliarity may play a role in HCPs' hesitation, which may decrease over time as seen elsewhere.^23^ Whereas quinine is an ancient medicine with which the population is very familiar, AS-AQ and artesunate are less known at certain facilities. In facilities where these antimalarial drugs are available (and thus known), preference for them is increased. In a parallel situation, a 2014 Ghanian study showed IV quinine having the same problem as artesunate currently has when HCPs persisted in prescribing chloroquine for severe malaria though it had been replaced by quinine in the national protocol, suggesting a simple persistence in old habits.^24^ Perhaps HCPs are more reluctant to use an unfamiliar medicine especially with sicker patients.

We see here a strong suggestion that consistent knowledge of a national protocol may be accompanied by HCP hesitancy about implementing it. The issue of HCP's acceptance of artemisinin-based therapy needs to be further addressed in order to assure solid implementation of this life-saving treatment for the country's number one cause of morbidity and mortality.

Though this study raises an important question, there are several limitations, including an inability to confirm the respondents' reported antimalarial availability, an inability to analyze the cluster effect of different sites, and a lack of a validated survey instrument. Lastly, the convenience sampling and lack of randomization of this study was not designed to be nationally representative, and the varying numbers of respondents from different health facilities may introduce selection bias. 

Regarding future avenues of study, a more expanded, randomized and nationally representative sample would be warranted, and this could also investigate to see if there are regional differences (for example, between varying levels of malaria endemicity). It is also necessary to explore HCPs' reasons for preferring another antimalarial drug to artemisinin-based therapy. In regards to artemisinin implementation, HCPs in other studies have cited doubts about efficacy, concerns about cost, and confusion in protocol implementation as reasons for non-compliance, though this reasons seems especially unexplored for IV artesunate.^12,23,25^ Additionally, it would be helpful to examine how these HCPs' preferences influence their actual practice, since practice is influenced by more factors than just preference.^26^

On the level of practical action, several ways forward are suggested by this data. Simply continuing to increase availability of oral ACTs and IV artesunate may be useful in augmenting exposure and acceptance of these antimalarials. Additional educational interventions may be useful, especially for the nursing workforce. However, this study suggests that knowing the recommendation for artemisinin-based therapy is not sufficient. Thus, educational interventions should include the rationale for the preference of artemisins, both in terms of increased efficacy and less side effects, in order to maximize acceptance and thus implementation. Lastly, the inclusion of HCP's preferences for such important antimalarial drugs could be useful to monitor by national malaria programs in order to continually assess further intervention needs.

It is unclear whether this preference of many HCPs for IV quinine in severe malaria is equally present in other malaria-endemic countries, but this may also warrant further study. Given the magnitude of the mortality benefit of IV artesunate over IV quinine for severe malaria, this could represent an urgent, high-impact area of research and intervention, since rapid improvement of protocol implementation could save numerous lives, as opposed to gradually waiting on the HCP population to become habituated to a newer therapy.^4,5^

## CONCLUSION

This study is the first in Burundi to evaluate HCP preferences towards artemisinin-based therapy versus quinine, which has revealed an important gap between knowledge of a therapeutic recommendation and practical preference, suggesting a problem of acceptance by health care professionals, which must be addressed for successful implementation of current malaria recommendations. In particular, the persistent preference for IV quinine over artesunate in severe malaria by many HCPs requires further investigation. This particular question is under-explored in the medical literature, and if present elsewhere, necessitates urgent research and intervention in order to efficiently apply this important life-saving advance in treatment. More comprehensive education about the advantages of artemisinin-based therapy and monitoring of HCP preferences by national malaria programs could also mitigate the effect of this implementation challenge.
